# Developmental Programming of the Metabolic Syndrome: Can We Reprogram with Resveratrol?

**DOI:** 10.3390/ijms19092584

**Published:** 2018-08-31

**Authors:** You-Lin Tain, Chien-Ning Hsu

**Affiliations:** 1 Departments of Pediatrics, Kaohsiung Chang Gung Memorial Hospital and Chang Gung University College of Medicine, Kaohsiung 833, Taiwan; tainyl@hotmail.com; 2 Institute for Translational Research in Biomedicine, Kaohsiung Chang Gung Memorial Hospital and Chang Gung University College of Medicine, Kaohsiung 833, Taiwan; 3 Department of Pharmacy, Kaohsiung Chang Gung Memorial Hospital, Kaohsiung 833, Taiwan

**Keywords:** developmental origins of health and disease (DOHaD), hypertension, metabolic syndrome, nitric oxide, nutrient-sensing signal, obesity, oxidative stress, renin-angiotensin system, resveratrol

## Abstract

Metabolic syndrome (MetS) is a mounting epidemic worldwide. MetS can start in early life, in a microenvironment that is now known as the developmental origins of health and disease (DOHaD). The concept of DOHaD also offers opportunities for reprogramming strategies that aim to reverse programming processes in early life. Resveratrol, a polyphenolic compound has a wide spectrum of beneficial effects on human health. In this review, we first summarize the epidemiological and experimental evidence supporting the developmental programming of MetS. This review also presents an overview of the evidence linking different molecular targets of resveratrol to developmental programming of MetS-related disorders. This will be followed by studies documenting resveratrol as a reprogramming agent to protect against MetS-related disorders. Further clinical studies are required in order to bridge the gap between animal models and clinical trials in order to establish the effective dose and therapeutic duration for resveratrol as a reprogramming therapy on MetS disorders from developmental origins.

## 1. Introduction

Metabolic syndrome (MetS) is a critical challenge for public health. Worldwide, two thirds of the deaths resulting from non-communicable diseases are mainly caused by MetS and related disorders [1]. MetS is a cluster of medical conditions including hypertension, obesity, dyslipidemia, non-alcoholic fatty liver disease (NAFLD), hyperglycemia and insulin resistance [2]. Despite the recent advances in medical and surgical treatment, there is still a global rising in the incidence and prevalence of MetS. Recent studies suggest that a suboptimal early-life environment can cause permanent changes of function and structure in specific organs that may increase the risk for developing MetS in later life [3,4,5], now being termed the developmental origins of health and disease (DOHaD) [6]. Several animal models have been developed to study the pathophysiology of developmental programming of MetS. The current models can be classified according to their nutritional, genetic, environmental and chemical factors [3,4,5]. The DOHaD concept offers a novel approach to prevent MetS through reprogramming, a strategy that shifts therapeutic intervention from adulthood to early life [7]. A number of pharmaceutical and dietary interventions have been examined as reprogramming strategies to prevent MetS and related disorders [3,4,5,7,8,9]. Among these, great attention has been given to resveratrol due to its beneficial effects on health [10,11,12]. Here, we first review the key themes on the developmental origins of MetS. This will be followed by presenting resveratrol supplementation as a possible reprogramming strategy to counter the growing epidemic of MetS and related disorders.

## 2. Epidemiological and Experimental Evidence for the Developmental Origins of Metabolic Syndrome

Several epidemiological studies indicate that fetuses exposed to suboptimal conditions increase the risk of MetS and related disorders. The first key evidence for developmental origins of MetS comes from the data gathered after severe famines, such as the Dutch Hunger Winter. Offspring born following famine exposure are prone to develop different phenotypes of MetS, such as obesity, diabetes and hypertension [13,14,15]. The risks for developmental programming of MetS have been assessed in a number of cohort studies, as reviewed elsewhere [7,16]. These risk factors for MetS of developmental origins involve a range of pre-, peri- and post-natal insults. Risks reported in these cohorts include: maternal malnutrition, maternal smoking exposure, maternal obesity, gestational hypertension, short-term breastfeeding, excessive postnatal weight gain and in utero exposure to endocrine-disrupting chemicals [7,16]. Further evidence comes from studies in twins, where associations have been reported between low birth weight and different phenotypes of MetS, including type 2 diabetes, hypertension and insulin resistance [17]. By last, a systematic review of 39 papers revealed that neonates born with low birthweight and experienced a rapid catch-up growth had a 79.6% increased risk for development of cardiovascular disease, a major complication of MetS [18]. All of these observations provide a link between the poor fetal and infantile environment and the risk of developing MetS and related disorders in later life.

So far, a number of nutritional, pharmacological and genetic models have been used to examine the mechanisms underlying MetS of developmental origins [3,4,5,19]. Different early-life insults can cause similar phenotypes of MetS, suggesting that common mechanisms can be involved in the development of MetS and related disorders. Although these common pathogenic mechanisms still remain inconclusive, certain programming mechanisms have been documented, including but not limited to, impaired nitric oxide (NO) system, endothelial dysfunction, oxidative stress, activation of the renin-angiotensin system (RAS), dysregulated nutrient sensing signals, autophagy and inflammation [4,7,9,19,20,21,22].

## 3. Health Benefits of Resveratrol against Metabolic Syndrome

Resveratrol, a polyphenolic compound (3,5,4′-trihydroxystilbene) produced by several plants, is a naturally occurring phytoalexin [23,24]. A growing body of evidence indicates that resveratrol has a broad range of beneficial effects on human health. The pleiotropic functions of resveratrol include the inhibition of platelet aggregation, improvement of endothelial function, anti-inflammatory and antioxidant properties, anticarcinogenic activity, anti-obesogenic and anti-atherosclerotic effects and restoration of NO bioavailability [10,23,24,25]. At the molecular level, multiple targets of resveratrol have been identified such as the silent information regulator 1 (SIRT1), 5′-adenosine monophosphate-activated protein kinase (AMPK), nuclear factor (erythroid-derived 2)-like 2 (Nrf2), nuclear factor-kappa B (NF-κB), estrogen receptor α (ERα), mammalian target of rapamycin (mTOR), peroxisome proliferator-activated receptor (PPAR), cyclooxygenase-2 (COX-2) and so on [25].

The works published in recent years confirm the link between molecular targets and signaling pathways of resveratrol and pathophysiology of MetS-related disorders. The decrease in endothelial nitric oxide synthase (eNOS) activity and NO bioavailability are crucial mechanisms underlying the endothelial dysfunction that occurs in MetS. Resveratrol has been reported to increase expression and activity of eNOS via activation of SIRT1 [26]. Asymmetric dimethylarginine (ADMA), an inhibitor of NOS, can be metabolized by dimethylarginine dimethylaminohydrolase-1 and -2 (DDAH1 and DDAH2) [27]. Resveratrol has been reported to restore DDAH activity and subsequently increase NO bioavailability [28]. Additionally, resveratrol blocks reactive oxygen species (ROS) formation via the down-regulated expression of NADPH oxidase subunits, like NOX [29]. Additionally, resveratrol enhances the antioxidant defense system by increasing the activity of several antioxidant enzymes such as superoxide dismutase (SOD) and glutathione peroxidase (GPx) [29]. These antioxidant effects of resveratrol are relevant to its molecular targets ERα and Nrf2. The activation of the RAS is crucial for the development of MetS and related disorders [30]. Resveratrol has been shown to suppress angiotensin II type 1 receptor (AT1R) expression through SIRT1 activation [31]. Furthermore, there are several molecular targets of resveratrol such as: AMPK, SIRT1, PPAR, PGC-1α and mTOR that can modulate nutrient-sensing signals [32]. Of note, the interplay between nutrient-sensing signals and oxidative stress plays an important role in cardiovascular programming, as we previously reviewed elsewhere [21]. Additionally, autophagy, a cellular catabolic process in which key organelles are transported to lysosomes for degradation, is dysregulated in MetS and related disorders [33]. AMPK can promote autophagy via inhibition of mTOR and many common inflammatory pathways have been implicated in the pathogenesis of MetS [3,4,5].

**Figure 1:**
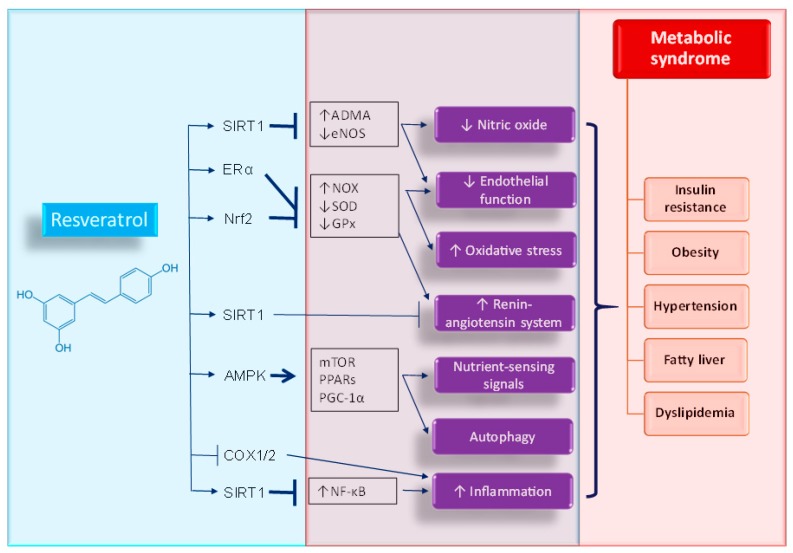
Schematic representation of beneficial effects and molecular targets of resveratrol against metabolic syndrome and related disorders. ↑ = increased. ↓ = decreased. Arrow = activation. T bar = inhibition.

Resveratrol inhibited the NF-κB inflammation pathway through activation of SIRT1 [34]. Independent of SIRT1, resveratrol is also a direct inhibitor of the cyclooxygenase enzymes 1- and -2 (COX1/2), which possibly contributes to its anti-inflammatory properties [25]. It is important to refer that the above-mentioned molecular targets and downstream signaling pathways of resveratrol point toward the pathophysiology of MetS and related disorders. Figure 1 is a graphic illustration of the beneficial effects and molecular target of resveratrol interrelated to MetS phenotypes.

Currently, a number of clinical trials show that resveratrol was well tolerated and had a beneficial influence in the disease biomarkers of MetS and related disorders [35,36], among these are diabetes, NAFLD, cardiovascular diseases and obesity. However, a wide range of therapeutic periods and doses of resveratrol (5 mg to 5 g) are presented in these clinical trials. Therefore, it is hard to determine the exact safety range and therapeutic window of resveratrol for specific patient populations. A systematic analysis of 21 studies with 681 subjects found that daily resveratrol consumption (≥300 mg/day) significantly reduced plasma total cholesterol, glucose levels and blood pressure in obese subjects. This daily intake of resveratrol also protected the subjects against cardiovascular diseases [37]. A meta-analysis of 283 subjects with type 2 diabetes reported that high-dose resveratrol (≥100 mg/day) supplementation improved the fasting plasma glucose level [38]. Nevertheless, other reports of meta-analysis fail to show the beneficial effects of resveratrol on lipid profile (*n* = 763 subjects) [39] and NAFLD (*n* = 158 subjects) [40].

In humans, resveratrol seems to have no obvious toxicity [41]. However, limited data are available regarding the effects of resveratrol supplementation during pregnancy on maternal and offspring’s health. Indeed, only two small studies showed that maternal resveratrol supplementation has a beneficial effect in pregnant women [42,43]. Although encouraging, the results of these clinical studies cannot per se directly establish a causal relationship between the particularly environmental insults and phenotypes of MetS. Hence is not a surprise that much of our knowledge on the types of early-life insults, developmental windows critical for developing MetS and mechanisms operating in resveratrol protection against MetS programming mainly come from studies in animal models. Here, we summarize some of the studies documenting the beneficial effects of resveratrol supplementation in animal models of developmental programming associated with MetS and related disorders (Table 1) [44,45,46,47,48,49,50,51,52,53]. Of note, resveratrol therapy will be narrowly restricted to those beginning prior to the onset of MetS-related phenotypes.

In the current review, limited information is available about the use of large animals to study the role of resveratrol on developmental programming of MetS-related disorders. Two reports in nonhuman primates shown that resveratrol supplementation during pregnancy improved maternal and placental phenotype with beneficial effects in the fetal liver of mothers exposed to Western diets [44,45]. Conversely, the work by Roberts and colleagues also reported that maternal resveratrol supplementation caused adverse effects on fetal pancreatic development [45]. Another study reported that maternal resveratrol supplementation stimulated placental DHA uptake, while maternal high-fat diet did not affect placental fatty acid profile [54]. However, there is not enough data regarding the fetal outcome after resveratrol supplementation and therefore, we excluded it from this review.

As shown in Table 1, rats are the most commonly used among the small animal models. Rats grow rapidly in childhood and reach sexual maturity after six weeks. In adulthood, one rat month is comparable to three human years [55]. Accordingly, Table 1 lists the metabolic outcomes evaluated in rodents with different ages, which allows calculations to extract the information that can be translated to humans of a specific age group. There are a number of early-life insults inducing MetS programming, such as maternal high-fat diet [46], prenatal hypoxia and postnatal high-fat diet [47,48,49], maternal plus post-weaning high-fructose diet [50], maternal plus post-weaning high-fat diet [51,52] and maternal low protein diet [53]. Although reprogramming effects of resveratrol on MetS-related phenotypes have been reported ranging rodents ranging from 3 to 16 weeks of age, there is a lack of substantial data regarding the long-term effects of resveratrol supplementation. It is clear from the preceding sections that some mechanisms determine the development of MetS-related disorders. In the current review, we focus on the reprogramming effects of resveratrol on MetS programming, and, for the sake of brevity, we have restricted the presented data to programming mechanisms related to resveratrol. Each of the above-mentioned potential mechanisms linking MetS of developmental origins to resveratrol will be discussed in the following section.

## 4. Potential Mechanisms of Resveratrol Supplementation on Metabolic Syndrome of Developmental Origins

### 4.1. Oxidative Stress

Oxidative stress, an imbalance between pro-oxidant and antioxidant factors, is considered a key mechanism that drives the developmental programming of MetS [3,4,5]. The developing fetus is highly vulnerable to oxidative stress damage due to its low antioxidant defense mechanisms [56]. As reviewed elsewhere [7,21], a great number of pre- and peri-natal adverse conditions can promote developmental programming attributed to oxidative stress, such as: maternal malnutrition, maternal diabetes, preeclampsia, prenatal hypoxia, maternal inflammation, prenatal glucocorticoid exposure and maternal high-fat diet. Conversely, resveratrol exhibits antioxidant properties against oxidative stress including scavenging ROS and enhancing antioxidant defensive actions [29]. Maternal resveratrol supplementation can protect adult offspring against low protein diet-induced oxidative stress, combined with mitigating MetS-related phenotypes [53]. Additionally, our previous work demonstrated that early post-weaning resveratrol treatment prevents the development of hypertension programmed by maternal plus post-weaning high-fructose diet, which is relevant for the reduction of oxidative stress damage [50]. However, antioxidant therapy is still not acknowledged as an unequivocal approach to control the global rise of MetS and related disorders. In the future, a better understanding of the mechanisms underlying the role of oxidative stress in the fetal programming of MetS is essential to establish the particular developmental window and organ-specific redox-sensitive signaling responsible for resveratrol as a reprogramming strategy for MetS of developmental origins [57].

### 4.2. Nutrient-Sensing Signals

Nutrient-sensing signals regulate cell metabolism and growth in response to variable nutrient levels. Therefore, nutrient-sensing signals have a unique role in the regulation of fetal growth and development. Several nutrient-sensing signals are also molecular targets of resveratrol, such as AMPK and SIRT1 [32]. Activation of AMPK or SIRT1 by resveratrol can affect PGC-1α activity to regulate the downstream expression of PPAR target genes [20,58]. Consequently, early resveratrol therapy can drive nutrient-sensing signals to regulate PPARs and their target genes and thereby reprogramming MetS-related programmed processes [20,59]. Additionally, early post-weaning resveratrol treatment activates AMPK and protects adult offspring against insulin resistance and hyperlipidemia in a combined prenatal hypoxia and postnatal high-fat diet rat model [49]. In agreement with this finding, we found that post-weaning high-fat diets induced hypertension and that reduced protein levels of phosphorylated AMPK2α in the offspring kidney were prevented by resveratrol therapy [52]. Similar to resveratrol, other AMPK activators have been reported to prevent the development of hypertension in a variety of fetal programming models [60]. These findings support the hypothesis that nutrient-sensing signals might be a common mechanism underlying the pathogenesis of MetS of developmental origins. Furthermore, many of these nutrient-sensing signals (e.g., AMPK, SIRT1 and mTOR) can either positively or negatively modulate the autophagic process. It is important to acknowledge that dysregulated autophagy is involved in MetS as well as in oxidative stress [33]. Since resveratrol tightly regulates the nutrient-sensing signals network, further studies are required to determine if maternal resveratrol supplementation has a role in the regulation of the nutrient-sensing signals to prevent fetal programming of MetS and which nutrient-sensing signal(s) can be considered the most promising target(s) for reprogramming.

### 4.3. Nitric Oxide

NO deficiency is a well-known pathogenetic mechanism of endothelial dysfunction that results from diverse cardiovascular risk factors such as hyperlipidemia, diabetes mellitus, obesity and hypertension [61]. NO deficiency can be caused by decreased substrate l-arginine availability, decreased NOS expression/activity, inactivation due to oxidative stress and inhibition by ADMA (an endogenous NOS inhibitor). Among the reasons for NO-ROS imbalance, increasing attention has been given to ADMA [27]. Indeed, increased plasma ADMA levels are associated with most of the clinical conditions associated with MetS such as hypertension, hypercholesterolemia, diabetes mellitus, obesity and NAFLD [27]. As reviewed elsewhere, impaired ADMA-NO pathway plays an important role in the pathogenesis of compromised pregnancies and fetal programming [62]. Conversely, a number of reprogramming strategies have been directed at influencing the balance of ADMA and NO in a wide range of models of cardiovascular programming [7,63]. Resveratrol can activate SIRT1 to reduce ADMA and increase NO bioavailability to protect against endothelial dysfunction [64]. Our previous study highlighted that resveratrol supplementation increased renal SIRT1 protein levels and reduced plasma ADMA levels protecting the offspring against hypertension induced by post-weaning high-fat diet [52]. It would be interesting to see whether resveratrol supplementation can also ameliorate other MetS-related phenotypes via mediating ADMA-NO pathway in different models of developmental programming.

### 4.4. Renin-Angiotensin System

The RAS is both a target of and contributor to MetS [30]. The RAS contains two opposite axes: the angiotensin converting enzyme (ACE)-angiotensin (Ang) II-AT1R classical axis mediated primarily by Ang II and the ACE2-angiotensin-(1-7)-Mas receptor axis mediated mainly by Angiotensin-(1-7) [65]. In contrast to ACE, ACE2 appears to control the expression levels of angiotensin II type 2 receptor (AT2R) and Mas receptor inhibiting the development of hypertension [65]. It is well known that over activation of the classical RAS leads to hypertension [65]. Additionally, hyperglycemia and insulin resistance have been demonstrated to activate RAS components (e.g., renin, ACE and AT1R) in specific cell types that mediate various phenotypes of MetS [30]. Conversely, early inhibition of the classical RAS has been shown to prevent the development of hypertension [66,67,68]. In addition to hypertension, RAS inhibition has been reported as a beneficial therapeutic approach to control impaired glucose levels, insulin tolerance and dyslipidemias in patients with MetS [30]. Recent evidence suggests that resveratrol can ameliorate most of the features of MetS and the beneficial effects of resveratrol treatment are commonly associated with down-regulation of the classical RAS axis and stimulation of the alternative RAS axis [69,70]. Nevertheless, little attention has been paid to explore the influence of resveratrol on the RAS components to prevent MetS of developmental origins. So far, only one report demonstrates that resveratrol could exert its protective effects on programmed hypertension induced by maternal post-weaning high-fat diet associated with decreased plasma Ang II level and increased plasma Ang (1-7) level [52]. Further studies are required to elucidate the detailed programming mechanisms underlying the modulation of RAS by resveratrol and whether these effects are protective against other MetS-related phenotypes using various programming models.

### 4.5. Others

There are other potential mechanisms related to MetS programming by which resveratrol might act: (1) by activating Nrf2, (2) by inhibiting NF-κB inflammation pathway and (3) by epigenetic regulation. Although Nrf2 activation and NF-κB inhibition have been reported to be protective in other developmental programming models [71,72,73], there is not enough data relating the reprogramming effects of resveratrol with MetS of developmental origins via regulation of Nrf2 and NF-κB. Additionally, epigenetic regulation has been considered an important mechanism underlying developmental programming [74]. Epigenetic processes, such as DNA methylation, covalent modifications of histones and non-coding RNAs, can induce changes in gene expression. Of note is that SIRT1 is a class III histone deacetylase (HDAC) family protein and resveratrol is an HDAC inhibitor [75]. HDACs have been reported to epigenetically regulate several genes belonging to the RAS [76]. Thus, it is speculated that resveratrol has epigenetic effects on RAS genes via HDAC dependent mechanisms.

## 5. Conclusions and Future Perspectives

Resveratrol supplementation as a reprogramming strategy against the development of MetS is a critical challenge and will become even more urgent in the near future as the growing epidemic of MetS and related disorders threaten humanity. The primordial studies in short-lived animal models, with controlled interventions across their life span provided key results revealing potential common mechanisms linking molecular targets of resveratrol to programming processes underlying MetS of developmental origins. Regardless of numerous beneficial effects of resveratrol, one major concern is its poor solubility and low intestinal absorption rate [77]. Thus, the development of resveratrol formulations with better pharmacologic properties remains a challenging task. Additionally, little is known regarding the dose-dependent effect of resveratrol in these programming models. Biotransformation of resveratrol is dependent on different species, doses, organs and disease status [78]. Furthermore, the metabolism of resveratrol also differs between humans and other species [78]. Thus, further translational research into the metabolism and pharmacokinetics of resveratrol is needed to validate and compare its safety and therapeutic potential between humans and other species. Importantly, clinical trials aiming to determine the effective dosage and therapeutic period of resveratrol as the reprogramming therapy for MetS and related disorders are urgently needed.

In the current review, the beneficial effects of resveratrol are attributed to several mechanisms that are known to interrelate with each other. Since resveratrol has a wide range of activities, it is critical to determine if maternal resveratrol therapy may generate off-target effects in adult offspring. During the preparation of the current review, we observed that multiple MetS-related phenotypes and target organs are not examined simultaneously in the same model. Additionally, the follow-up periods after the cessation of resveratrol supplementation in most cited reprogramming studies were relatively short. We, therefore, must determine the long-term programming effects of resveratrol in different programming models and clarify whether there is an organ-specific response. Current evidence of the reprogramming effects of resveratrol is still in its infancy. Further research and more detailed clinical studies are required in order to ensure the beneficial effects of resveratrol on MetS-related disorders and for ascertaining the critical window, drug dose and therapeutic duration for reprogramming therapy.

## Figures and Tables

**Table 1:** Relevant studies on resveratrol supplementation as a reprogramming strategy in animal models of fetal programming associated with metabolic syndrome-related phenotypes.

Dose and Period of Resveratrol Supplementation	Animal Models	Gender/Species	Group Size	Age at Evaluation	Beneficial Effects on Offspring	Ref.
0.37% *w*/*w* resveratrol in diet between 3 months before the breeding and gestational day 130	Maternal Western-style diet	Japanese macaques	*N* = 6	Gestational day 130	Restored the loss of fetal islet vascularity	[44]
0.37% *w*/*w* resveratrol in diet between 3 months before the breeding and gestational day 130	Maternal Western-style diet	Japanese macaques	*N* = 6	Gestational day 130	Reduced lipid deposition in the fetal liver	[45]
Resveratrol (50 mg/L) in drinking water during pregnancy and lactation	Maternal high-fat diet	Male and female Wistar rats	*N* = 4–6	3 weeks	Attenuated hyperglycemia, obesity and hyperlipidemia	[46]
Resveratrol (4 g/kg of diet) between 3–12 weeks of age	Prenatal hypoxia and postnatal high-fat diet	Male SD rats	*N* = 6	12 weeks	Prevented hypertension	[47]
Resveratrol (4 g/kg of diet) between 3–12 weeks of age	Prenatal hypoxia and postnatal high-fat diet	Male and female SD rats	*N* = 10	12 weeks	Attenuated insulin resistance in males	[48]
Resveratrol (4 g/kg of diet) between 3–12 weeks of age	Prenatal hypoxia and postnatal high-fat diet	Male SD rats	*N* = 6	12 weeks	Attenuated insulin resistance and hyperlipidemia	[49]
Resveratrol (50 mg/L) in drinking water from weaning to three months of age	Maternal plus post-weaning high-fructose diet	Male SD rats	*N* = 8	12 weeks	Prevented hypertension	[50]
0.2% *w*/*w* resveratrol in diet during pregnancy and lactation	Maternal plus post-weaning high-fat diet	Male C57BL/6 J mice	*N* = 10	14 weeks	Prevented obesity and hyperlipidemia	[51]
0.5% resveratrol in drinking water between 2 and 4 months of age	Maternal plus post-weaning high-fat diet	Male SD rats	*N* = 8–10	16 weeks	Prevented hypertension	[52]
Resveratrol (20 mg/kg/day) via oral gavage during pregnancy	Maternal low protein diet	Male and female Wistar rats	*N* = 7–14	16 weeks	Attenuated obesity and insulin resistance	[53]

Studies tabulated according to offspring age at evaluation; SD rats = Sprague-Dawley rats.
